# Rethinking tractography and neuroanatomy: does image resolution hold the key?

**DOI:** 10.1007/s00429-025-03025-0

**Published:** 2025-10-23

**Authors:** Tim B. Dyrby, Laurent Petit, Christian Beaulieu

**Affiliations:** 1 Danish Research Centre for Magnetic Resonance, Department of Radiology and Nuclear Medicine, Copenhagen University Hospital Amager and Hvidovre, Hvidovre, Denmark; 2 Department of Applied Mathematics and Computer Science, Technical University of Denmark, Kongens Lyngby, Denmark; 3 University of Bordeaux, CNRS, IMN, UMR 5293, Bordeaux, 33000 France; 4 IRP/LIA OpTeam, CNRS Biologie et Université de Bordeaux, France - Université de Sherbrooke, Canada, Bordeaux, France; 5 Departments of Radiology and Diagnostic Imaging & Biomedical Engineering, University of Alberta, Edmonton, AB Canada

## Abstract

The structural brain connectome spans multiple length scales with varying complexity, from major commissural, association, and projection pathways down to much smaller pathways within brain regions. Diffusion MRI is often acquired for whole-brain coverage with limited spatial resolution for tractography purposes. How does this ’one size fits all’ approach impact tractography and diffusion quantification, and do we need to care? Here, we discuss the concept of image resolution and define the structural image resolution limit, representing the resolution threshold at which a given dMRI setup can reliably resolve anatomical structures.

## Introduction

The white matter pathways in the brain form a complex structural connectome, spanning a wide range of length scales. Major pathways, such as commissural, association, and projection tracts, consist of smaller parallel-running fibers that diverge to reach their target regions. While the size of these major pathways allows for rough visual inspection in clinical Magnetic Resonance Imaging (MRI) scans, smaller tracts may be indistinguishable or have erroneous diffusion metrics due to partial volume effects (PVE) closely related to the acquired image resolution. This highlights a dilemma not directly related to image resolution per se, but rather to the relationship between voxel size and the size of anatomical structures. This defines a structural resolution limit, which is analogous to the Nyquist sampling theorem stating that the sampling frequency must be at least twice the frequency of the signal to reconstruct it accurately. In our case, the sampling frequency is determined by the voxel size, while the anatomical geometry represents the “waveform” we aim to reconstruct.

Indeed, diffusion MRI of fiber orientation distribution (dMRI FOD) can reveal multiple directions within crossing fiber voxels, which can be seen as a form of PVE suppression with limited resolution. Similarly, interpolating raw dMRI data followed by fiber reconstruction shows improved contrast in, for example, Fractional Anisotropy (FA) maps where non-parallel pathways interface (i.e., crossings) (Dyrby et al. 2014). Track Density Imaging also uses interpolation to reduce PVE. In line with these approaches, whole-brain streamline-based tractography is performed using standard deterministic tractography methods, where the stepwise-updated fiber orientation is determined by interpolating fiber orientations from neighboring voxels at the original image resolution. Here, the key idea is that instead of reporting the voxel-wise streamline count (density) at the original image resolution, it is computed and outputted at the higher interpolated resolution (Calamante et al. 2010). However, interpolation does not necessarily increase anatomical information - i.e., it does not guarantee the ability to reveal finer sub-voxel structures. The explanation is that an FOD can be viewed as a summation of the micro-domain FOD environment within the voxel. Each micro-domain FOD spatially outlines the local FOD along axons and is so small that they do not detect crossing fibers (Kjer et al. 2025). When summing all micro-domain FODs into a single voxel-based FOD, the two will be consistent in terms of angular information, but the spatial information of the micro-domains is lost. This spatial information cannot be recovered retrospectively. Hence, while clinical dMRI with limited image resolution in relation the finer pathways is widely used to study the brain’s structural connectome, concerns remain about the precision of tractography, particularly in bottleneck regions where pathways cross or kiss.

Interestingly, pathways are spatially organized, crossing or interfacing as distinct fascicles or laminar structures at specific angles, rather than as a chaotic interweaving of axons. This suggests that image resolution may be less critical than addressing the methodological challenges of tractography. However, does this hold when considering that pathways span anatomical scales ranging from millimeters to sub-millimeters?

## Did you know (I) that structural connectome analysis may perform better at a lower spatial resolution?

Can structural connectivity metrics improve not by increasing but rather by lowering diffusion MRI image resolution? Surprisingly, that is what was reported in (Ambrosen et al. 2020) when validating structural connectomes against the tracer-based connectivity matrix from (Markov et al. 2014) using ex vivo monkey brains. Comparing resolutions of 0.5 mm, 1.0 mm, and 2.0 mm isotropic, improvement in tracts was observed at the two lower resolutions (note: scaled to human brains with a volume ratio of 13, these would correspond to 1.2 mm, 2.3 mm, and 4.7 mm).

**Fig. 1:**
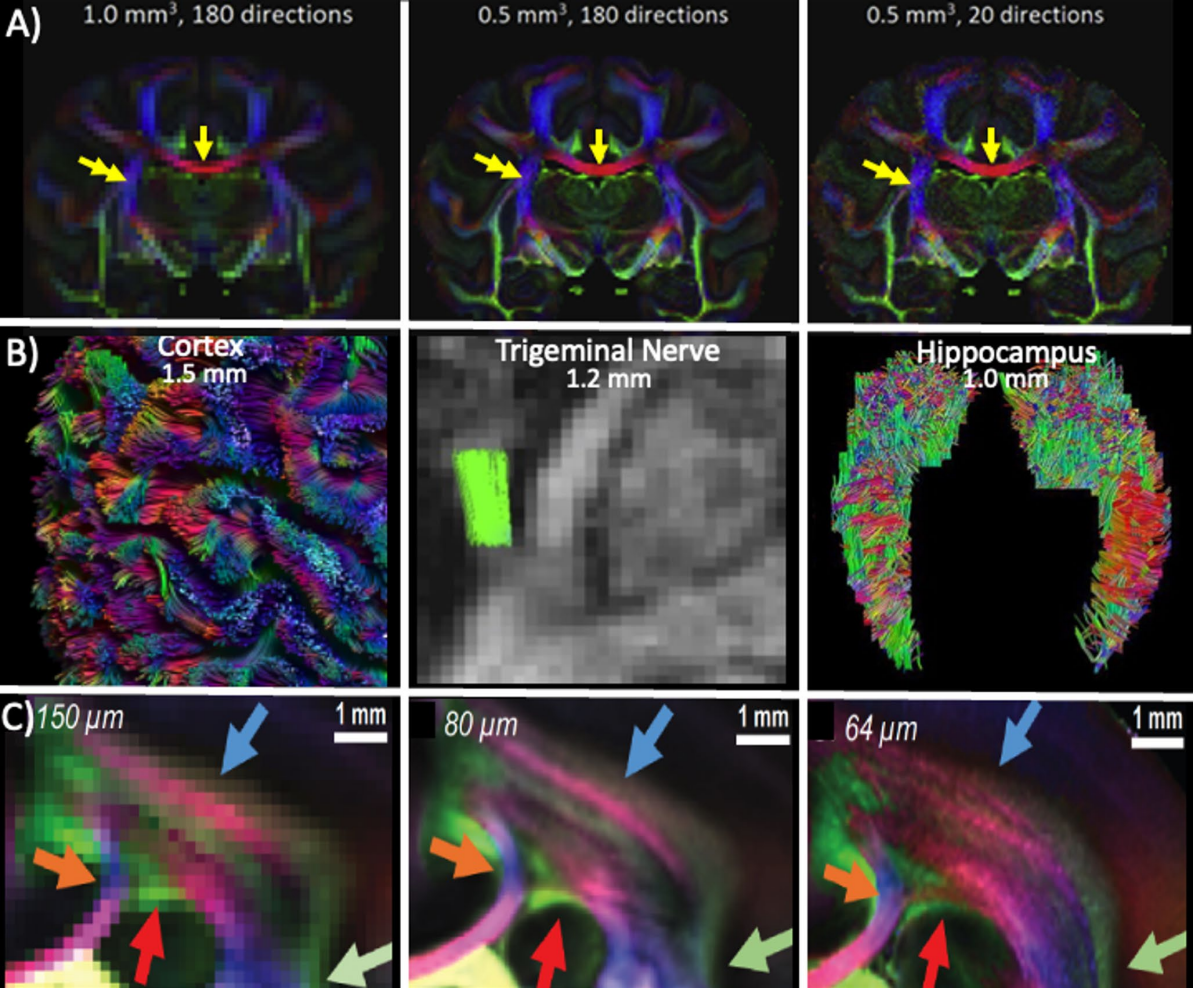
The representation of anatomical structure size is related to the image resolution demonstrating the importance of considering the structural resolution limit. **A** Ex vivo whole-brain Rhesus macaque monkey brain dMRI scanned at different image resolutions and number of diffusion encoding directions. The major pathways Corpus Callosum (arrow) and the corticospinal tract (double arrow) are well represented at the lower image resolution independent of the number of measured diffusion encoding directions (Modified Fig. 2 from (Ambrosen et al. 2020). **B** High-resolution acquisition beyond 2 mm isotropic resolution can be helpful for tractography of small/thin/complex regions such as the human cerebral cortex at 1.5 mm isotropic (image courtesy of A. Leemans, Utrecht University), trigeminal nerve at 1.2 mm isotropic (nerve portion shown in green overlay on FA map (Danyluk et al. 2021), and hippocampus at 1.0 mm isotropic resolution. **C** Color-coded fractional anisotropy map reveals the intricate organization of brain fiber crossings for a given image resolution. Coronal slices with zoomed-in views showing the corona radiata of a postmortem marmoset brain acquired at increasing spatial resolution. The color-coded FA map shows fiber orientations. Colored arrows highlight areas that benefit from the use of higher spatial resolution. (modified from (Liu et al. 2020).

A visual inspection of the color coded FA maps in Fig. 1A reveals expected blurring at lower resolutions. Yet, major fiber bundles such as the corticospinal tract and corpus callosum remain clearly distinguishable, which is key to understanding this effect. The explanation lies in how tractography works: it does not require full cross-sectional resolution but instead follows voxel-by-voxel anisotropy to draw streamlines.

In structural connectomes, the focus is typically on whether streamlines successfully connect two cortical regions rather than the exact pathway cross-section. Connectivity “strength” (streamline count) is biased by path length dependency: the more voxels a streamline must traverse, the lower its probability of survival, leading to apparent “weaker” connectivity (Donahue et al. 2016; Liptrot et al. 2014; Dyrby et al. 2014, 2025; Jones 2010).

Three factors influence connectivity strength: (i) image resolution, (ii) pathway cross-section area, and (iii) pathway length in voxels. If lower resolution increases connectivity, it suggests that only major fiber pathways—those large enough to be fully resolved—are preserved according to the structural resolution limit. Lower image resolution acts as a filter, suppressing sensitivity to smaller pathways via partial volume effects (PVE) (Dyrby et al. 2014), where larger pathways dominate the fiber orientation distribution and exhibit straighter trajectories. Smaller or less straight pathways are biased in the FOD, as fiber orientation at the subvoxel scale loses spatial trajectory information (Kjer et al. 2025). Additionally, fewer voxels along a pathway reduce path length dependency.

Thus in structural connectome analysis, a relative image resolution should be considered based on the cross-sectional area of the pathway of interest. If the goal is to reduce path length dependency and improve signal-to-noise ratio (SNR), the lowest possible resolution that still adequately resolves the major pathways of interest should be prioritized.

### Did you know (II) that high spatial resolution is important for small brain regions and there are strategies to enable clinically relevant scan times?

Human in vivo diffusion MRI protocols for tractography often prioritize high b-values, multiple shells, and numerous diffusion directions, while spatial resolution and image quality are compromised to maintain SNR and minimize scan time relative to the number of images. This ‘low-resolution’, whole-brain approach (e.g., 2 mm isotropic or worse in humans) poses challenges for smaller brain regions and leads to a loss of anatomical specificity due to PVE and bias in the diffusion metrics. Unlike in DYK (I) above, where diffusion properties for major pathways are the focus, here, precise representation of smaller anatomical structures matters.

Improving the spatial resolution of in vivo dMRI is challenging, as SNR scales with voxel volume and the square root of scan time. For example, keeping all other parameters the same, reducing voxel size from 2 × 2 × 2 mm^3^ (8 mm³) to 1 × 1 × 1 mm^3^ (1 mm³) would require 64× longer scan times, making it impractical. However, advances in MRI hardware - higher static fields, phased-array radiofrequency coils, multiband excitation, and stronger gradients - offer ways to mitigate these limitations. Trade-offs are inherent in MRI protocol design so one can restrict brain coverage to only brain regions of interest which can optimize scan time for clinical applications, and explore lower b-values to improve SNR when estimating, for example, diffusion tensor imaging (DTI) parameters. The Human Connectome Projects (HCP) has successfully leveraged recent hardware advancements in 3 T MRI to gain higher image resolution. While whole-brain acquisitions at 1.25 mm isotropic (2 mm³ voxel volume) still require relatively long scan times given the focus on many b-value shells and directions (Fan et al. 2022), 1.5 mm isotropic (3.4 mm³ voxel volume) has been achieved with more feasible scan times in developmental and aging HCP cohorts (Harms et al. 2018). Higher spatial resolution enables better examination of smaller white matter tracts, such as superficial white matter, short association fibers, fornix, cerebellum, and brainstem.

Higher-resolution diffusion MRI tractography of three small, thin, and/or complex regions (e.g. cortex, trigeminal nerve, hippocampus) are shown in Fig. 1B, all using standard single-shot EPI at 3 T, but each with a customized acquisition strategy to enable a workable spatial resolution in a clinically relevant scan time. In these examples, one compromise was a focus on single shells with lower b-values and/or limited gradient directions, inherently with an emphasis on DTI analysis. The cortex is only ~ 1–5 mm thick with a complex folding pattern and exhibits primarily radial diffusion (i.e., primary eigenvector perpendicular to the cortical surface), which remains detectable at 1.5 mm isotropic resolution in a 3.5 min scan (Little and Beaulieu 2021). The trigeminal nerve is a small peripheral nerve (~ 2.7 mm diameter) surrounded by cerebrospinal fluid which can deleteriously affect tractography and DTI metric quantification, necessitating higher than usual resolution (1.2 mm isotropic) over a limited number of 13 slices oriented along the length of the nerve with a short scan time of 3.5 min (Danyluk et al. 2021). Again, this scan only acquires a single shell for DTI which is fine for an isolated tract without crossing fibers. The hippocampus, a heterogeneous and convoluted structure, is well-resolved at 1 mm isotropic resolution in a 6 min scan (Treit et al. 2018), which would enable regional identification of lesions that do not affect the entire hippocampus. In this case, a low b-value (500 s/mm²) was used to limit signal loss relative to typically used higher b values, but it also reduced the diffusion-sensitizing gradient duration and hence echo time (TE), further enhancing SNR. Additionally, instead of the standard whole brain coverage, a focused field-of-view including 20 slices aligned along the hippocampus was used to minimize repetition time (TR), allowing more time for averaging and additional diffusion directions to boost SNR. Tractography in gray matter regions such as hippocampi is emerging, but may require more complex protocols in addition to higher spatial resolution. Other brain regions could also benefit from custom dMRI scans.

Thus, standard single-shot EPI enables high-resolution diffusion MRI within clinically relevant scan times at 3 T when using modern MRI hardware. However, region-specific acquisition strategies for smaller structures are crucial rather than whole-brain-attempt-to-fit-all protocols.

### Did you know (III) that a simple color-coded fractional anisotropy map reveals the intricate organization of brain fiber crossings?

The study by (Liu et al. 2020) highlights that higher spatial resolution in dMRI acquired on postmortem marmoset brains significantly enhances the ability to differentiate fibers from distinct bundles, even in basic color-coded FA maps, the so-called RGB maps. For instance, in the corona radiata (Fig. 1C), an image resolution of 150 μm reveals a general crossing between greenish association pathways and pinkish/reddish commissural pathways. However, at 64 μm resolution, the underlying organization of these fibers becomes much clearer, revealing an intricate structure of interlaced sub-bundle layers of two distinct WM pathways that are more defined and distinguishable. These higher resolution, color-coded FA maps offer crucial insights into the topology of crossing fibers. Rather than appearing as a coarse or random intersection at lower image resolutions, the callosal and association fibers in the corona radiata reveal an organized laminar crossing pattern at 64 μm. These sublayers exhibit dominant bright colors in the RGB map, reflecting their systematic spatial arrangement. Such structural precision demonstrates that these pathways adhere to an elegant, spatially constrained organization, even within regions where fibers must navigate through bottlenecks or crossing regions.

It is important to note, however, that a sub-bundle layer observed at 64 μm resolution does not consist of fibers with a single direction (Schilling et al. 2016). Additional orientations and finer-scale complexities that contribute to the topology of these regions are uncovered as spatial resolution approaches the diameter of individual axons. Actually, Kjer et al. bridged anatomical length scales within a volume of a MRI voxel of 0.5^3^ mm^3^ down to individual axons at a 75^3^ nm^3^ image resolution using X-ray nanoholotomography (XNH) synchrotron imaging (Kjer et al. 2025). Submicrometer tractography was applied to show how white matter is organized as laminar structures of different thickness across anatomical length scales and image resolutions - observed both in aligned pathways such as Corpus Callosum and in the Centrum Semiovale region including crossing fibers (Kjer et al. 2025; Liu et al. 2020). The inclination angle between layers determine if they belong to the same pathway i.e., aligned or different if crossing (grid like). While dMRI based tractography has advanced significantly to detect crossing fibers, they remain blind to certain features of axonal architecture, such as collateral branches that allow axons to connect to multiple targets (Economo et al. 2016) (but see (Lundell et al. 2011) about collateral fibers observed in tractography in the cervical spinal cord. Even so, the presence of distinct sub-layering within dense crossings highlights the precise and systematic organization of white matter, dispelling the notion of chaotic intermingling and instead revealing a meticulously structured and highly orchestrated architectural framework.

The implications of these advances extend beyond mere visualization. At submillimeter resolution scales, tractography may tract pathways with unprecedented accuracy, allowing us to uncover connections that were previously inaccessible. This level of detail has the potential to revolutionize our understanding of neural circuits in both health and disease. Submillimeter tractography in preclinical studies could, for instance, reveal microstructural alterations in conditions like multiple sclerosis, traumatic brain injury, or neurodegenerative disorders, offering novel biomarkers for early detection and targeted treatment strategies. Additionally, such fine-scale tractography may elucidate how structural connectivity evolves with development or adapts during rehabilitation and recovery.

Interestingly, despite being based on the tensor model which cannot resolve crossing fibers, high-resolution color-coded FA maps alone could have profound implications. At a sufficiently high resolution, these maps provide an intuitive and accessible means of visualizing interlaced sub-bundle layers that would appear as mere ‘crossing fibers’ at lower resolutions, offering valuable insights into structural connectivity without the need for complex computational modeling. For instance, clinicians and researchers could use color-coded FA maps to identify subtle disruptions in the organization of sub-bundle layers caused by injury, disease, or aging. Furthermore, these maps could guide tractography algorithms by offering a clearer understanding of the dominant orientations within complex regions, improving the accuracy and reliability of downstream analyses.

## Conclusion

The short answer to whether image resolution holds the key is: Yes!

Both diffusion MRI-based tractography and microstructural methods, along with acquisition techniques and scanner hardware, have undergone tremendous advancements over the past two decades. Our three *Did-You-Know* reflection topics illustrate that image resolution is fundamental to rethinking tractography and improving its performance and precision.

However, as a research field, we must avoid tunnel vision by focusing solely on absolute image resolution. Instead, we should consider *relative image resolution* - i.e., resolution in relation to anatomical structure sizes. Absolute image resolution refers to the resolution offered by the scanner, while relative image resolution describes how structures, such as pathways of a given size, are spatially represented.

Thus, the relative image resolution defines a *structural image resolution limit*, representing the resolution threshold at which a given dMRI setup can reliably resolve anatomical structures - like its cross-sectional area. This limitation is linked to the well-known Nyquist theorem in signal processing, highlighting the need to balance resolution, signal quality, and anatomical precision in tractography. Hence, quantification of pathways geometries such as their cross sectional area must be accounted for in relation to the image resolution.

## Data Availability

No datasets were generated or analysed during the current study.

## References

[CR1] Ambrosen KS, Simon F, Eskildsen M, Hinne K, Krug H, Lundell MN, Schmidt, Marcel AJ, van Gerven M, Mørup, Dyrby TB (2020) Validation of structural brain connectivity networks: the impact of scanning parameters. NeuroImage 204:116207 31539592 10.1016/j.neuroimage.2019.116207

[CR2] Calamante F, Tournier J-D, Jackson GD, and Alan Connelly (2010) Track-Density imaging (TDI): Super-Resolution white matter imaging using Whole-Brain Track-Density mapping. NeuroImage 53(4):1233–1243 20643215 10.1016/j.neuroimage.2010.07.024

[CR3] Danyluk H, Sankar T, Beaulieu C (2021) High Spatial resolution nerve-Specific DTI protocol outperforms Whole-Brain DTI protocol for imaging the trigeminal nerve in healthy individuals. NMR Biomed 34(2):e4427 33038059 10.1002/nbm.4427

[CR4] Donahue CJ, Stamatios N, Sotiropoulos S, Jbabdi M, Hernandez-Fernandez TE, Behrens TB, Dyrby T Coalson, et al (2016) Using diffusion tractography to predict cortical connection strength and distance: A quantitative comparison with tracers in the monkey. J Neuroscience: Official J Soc Neurosci 36(25):6758–6770 10.1523/JNEUROSCI.0493-16.2016 PMC4916250 27335406

[CR5] Dyrby TB, Fieremans E, Rheault F, Anderson AW, Palombo M, Sarubbo S, Neher P, Schilling KG (2025) Tractography validation part 1: foundations, numerical simulations, and phantom models. In: Handbook of diffusion MR tractography. Elsevier, pp. 485–509

[CR6] Dyrby TB, Lundell H, Burke MW, Reislev NL, Paulson OB, Ptito M, and Hartwig R. Siebner (2014) Interpolation of diffusion weighted imaging datasets. NeuroImage 103(December):202–213 25219332 10.1016/j.neuroimage.2014.09.005

[CR18] Economo MN, Clack NG, Lavis LD, Gerfen CR, Svoboda K, Myers EW, Chandrashekar J (2016) A platform for brain-wide imaging and reconstruction of individual neurons. Elife 5:e10566. 10.7554/eLife.10566 10.7554/eLife.10566 PMC4739768 26796534

[CR7] Fan Q, Eichner C, Afzali M, Mueller L, Tax CMW, Davids M, Mahmutovic M et al (2022) Mapping the human connectome using diffusion MRI at 300 mT/m gradient strength: methodological advances and scientific impact. NeuroImage 254(118958):118958 35217204 10.1016/j.neuroimage.2022.118958 PMC9121330

[CR8] Harms MP, Leah H, Somerville BM, Ances J, Andersson DM, Barch M, Bastiani SY, Bookheimer et al (2018) Extending the human connectome project across ages: imaging protocols for the lifespan development and aging projects. NeuroImage 183(December):972–984 30261308 10.1016/j.neuroimage.2018.09.060 PMC6484842

[CR9] Jones DK (2010) Challenges and limitations of quantifying brain connectivityin vivowith diffusion MRI. Imaging Med 2(3):341–355

[CR10] Kjer HM, Andersson M, He Y, Pacureanu A, Daducci A, Pizzolato M, Salditt T et al (2025) Bridging the 3D geometrical organisation of white matter pathways across anatomical length scales and species. eLife 13. 10.7554/eLife.94917 10.7554/eLife.94917 PMC11870653 40019134

[CR11] Liptrot MG, Sidaros K, Dyrby TB (2014) Addressing the Path-Length-Dependency confound in white matter tract segmentation. PLoS ONE 9(5):e96247 24797510 10.1371/journal.pone.0096247 PMC4010423

[CR12] Little G, Beaulieu C (2021) Automated cerebral cortex segmentation based solely on diffusion tensor imaging for investigating cortical anisotropy. NeuroImage 237(118105):118105 10.1016/j.neuroimage.2021.118105 33933593

[CR13] Liu C, Ye FQ, Newman JD, Szczupak D, Tian X, Yen CC-C, Majka P et al (2020) A resource for the detailed 3D mapping of white matter pathways in the marmoset brain. Nat Neurosci 23(2):271–280 31932765 10.1038/s41593-019-0575-0 PMC7007400

[CR14] Lundell H, Nielsen JB, Ptito M, Dyrby TB (2011) Distribution of collateral fibers in the monkey cervical spinal cord detected with Diffusion-Weighted magnetic resonance imaging. NeuroImage 56(3):923–929 21352926 10.1016/j.neuroimage.2011.02.043

[CR15] Markov NT, Ercsey-Ravasz MM, Ribeiro Gomes AR, Lamy C, Magrou L, Vezoli J, Misery P et al (2014) A weighted and directed interareal connectivity matrix for macaque cerebral cortex. Cereb Cortex (New York N Y : 1991) 24(1):17–36 10.1093/cercor/bhs270 PMC3862262 23010748

[CR16] Schilling K, Janve V, Gao Y, Stepniewska I, Landman BA, Adam WA (2016) Comparison of 3D orientation distribution functions measured with confocal microscopy and diffusion MRI. NeuroImage 129 (April):185–97 10.1016/j.neuroimage.2016.01.022 PMC4803575 26804781

[CR17] Treit S, Steve T, Gross DW, and Christian Beaulieu (2018) High resolution in-Vivo diffusion imaging of the human hippocampus. NeuroImage 182(February):479–487 29395905 10.1016/j.neuroimage.2018.01.034

